# Buccal soft tissue lipoma in an adult Nigerian: a case report and literature review

**DOI:** 10.1186/1752-1947-2-382

**Published:** 2008-12-13

**Authors:** Adeyi A Adoga, Tonga L Nimkur, Agabus N Manasseh, Godwin O Echejoh

**Affiliations:** 1 Department of Surgery (Otorhinolaryngology unit), Jos University Teaching Hospital, PMB 2076, Jos, Plateau State, Nigeria; 2 Department of Pathology, Jos University Teaching Hospital, PMB 2076, Jos, Plateau State, Nigeria

## Abstract

**Introduction:**

Lipomas are benign mesenchymal neoplasms composed of mature adipocytes, usually surrounded by a thin fibrous capsule. They are uncommon intra-oral tumors with 1% to 4% occurring in this region. The literature is scanty on lipomas occurring in the buccal soft tissue, especially in our environment.

**Case presentation:**

We present a case of a 35-year-old woman of the Tiv ethnic group of Nigeria who presented with a slow growing left cheek swelling that was treated by intra-oral local excision.

**Conclusion:**

The purpose of this report is to highlight the existence of this rare but not uncommon disease even in our environment and to emphasize that a high index of suspicion is needed in making a diagnosis. Surgical excision as treatment is associated with an excellent outcome.

## Introduction

Lipomas are benign mesenchymal neoplasms composed of mature adipocytes, usually surrounded by a thin fibrous capsule [1]. They are slow growing, painless masses with the subcutaneous and retroperitoneal spaces which contain abundant fat being the most common sites [2]. Fifteen to 20% occur in the head and neck region [3]. However, only 1% to 4% occur in the oral cavity [3,4].

Adequate surgical excision in order to prevent recurrence is the treatment of choice [1,5]. We report a case of a 35-year-old woman of the Tiv ethnic group of Nigeria who presented with a slow growing left cheek swelling that was treated by intra-oral local excision.

Although an isolated case of buccal soft tissue fibrolipoma has been reported in our environment [2], this paper presents the first case of buccal soft tissue lipoma seen in our institution and brings to the fore the existence of this rare disease.

## Case presentation

A 35-year-old housewife of the Tiv ethnic group in Nigeria was referred to our Ear, Nose and Throat clinic by family physicians with a 6-year history of a slowly progressive, painless left cheek swelling not preceded by trauma and not associated with fever, weight loss or any other otorhinolaryngological symptoms. Examination revealed a 6 cm by 6 cm non-tender doughy mass in the left cheek with no overlying skin changes. Slipping sign was not demonstrable and there was no bruit over this mass. The intra-oral mucosa over the mass appeared normal. A provisional diagnosis of buccal soft tissue lipoma was made with epidermoid cyst as a differential diagnosis.

Imaging using ultrasonography revealed a fairly well circumscribed echogenic mass in the left cheek measuring 1.67 cm by 1.23 cm with no evidence of neovascularization noted within. On this premise, the radiologist made an assessment of lipoma. A computerized tomographic scan was not done because the patient could not afford to pay for it. Other investigations performed included full blood count, serum urea and electrolyte, and urinalysis which were all within normal limits.

**Figure 1:**
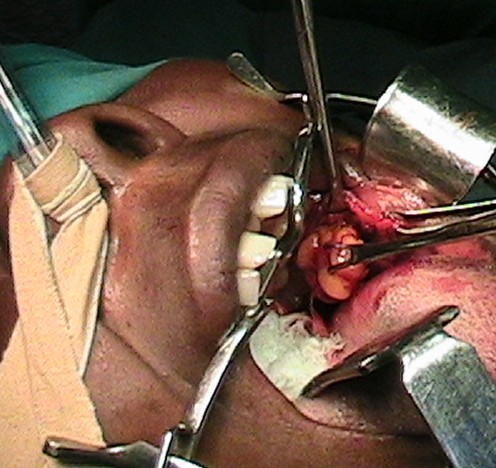
Lipoma being excised via an intra-oral incision.

**Figure 2:**
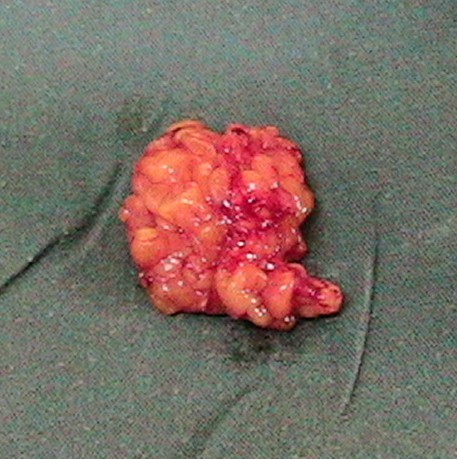
Excised lipoma.

She was prepared for and had excision under general anesthesia via naso-endotracheal intubation. During surgery, the mass was approached intra-orally by a transverse 5 cm linear incision made in the mucous lining over it (Figure 1). The 4 cm by 4 cm irregular yellowish mass (Figure 2) was carefully excised and the wound closed using a chromic 3/0 suture.

**Figure 3:**
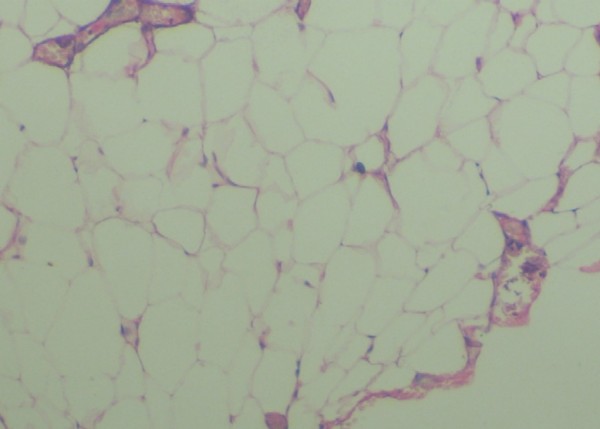
Photomicrograph showing aggregates of mature adipocytes with large clear cytoplasms and eccentric nuclei. Hematoxylin and eosin stain ×20.

Microscopic examination of the excised soft tissue mass revealed sheets of mature adipocytes containing large clear cytoplasms and eccentric nuclei with inconspicuous vascularity and no evidence of cellular atypia or metaplasia (Figure 3). These features are consistent with a classical diagnosis of a lipoma.

Postoperatively, she was placed on ciprofloxacin, ibuprofen and vitamin C tablets with oral saline mouth wash after meals. She was discharged in good condition on the 5^th ^postoperative day and has remained free of any symptoms for over 36 months of follow-up.

## Discussion

Lipomas are adipose mesenchymal neoplasms that rarely occur in the oral cavity with a 1% to 4% reported occurrence in this region [3,4]. The peak incidence age for lipoma is 40 years and above [5]. Generally, their prevalence does not differ with gender, although a male predilection has been recorded [6].

In the oral cavity, the most common sites are the cheek, tongue, palate, mandible and lip where lipomas occur as sessile or encapsulated masses [2]. The etiology is unknown. However, it is thought that trauma may trigger proliferation of fatty tissue and cause a lipoma [7].

The classification for benign lipomas includes the following: classic lipoma; lipoma variants (for example angiolipoma, chondroid lipoma, myolipoma, spindle cell lipoma); hamartomatous lesions; diffuse lipomatous proliferations and hibernoma [8].

Oral lipomas are slow growing tumors and patients commonly present with a well circumscribed mass that has been growing for several years [9]. Our patient reported a lesion of 6 years duration.

Clinically, they present as soft and compressible masses with doughy consistency which are well defined clinically and radiologically using ultrasonography and computerized tomographic scan [10] and more recently, using magnetic resonance imaging [11]. In some cases, they can present as fluctuant nodules [12]. Because of the diverse modes of presentation, some other lesions should be considered in the differential diagnosis and these include oral lymphoepithelial cysts, epidermoid and oral dermoid cysts [13].

Unlike oral lipomas, lymphoepithelial cysts are found in the floor of the mouth, soft palate and mucosa of the pharyngeal tonsil [14]. Although oral dermoid and epidermoid cysts can occur in other sites of the oral mucosa [4], they typically occur on the midline of the floor of the mouth [15].

Adequate surgical excision is the treatment for oral lipomas [1,5]. The surgical approach is dependent on the site of the tumor and the proposed cosmetic result. Our patient's lipoma was approached intra-orally with excellent outcome.

Microscopically, it is difficult to differentiate between normal adipose tissue and lipomas, therefore, a clinician sending a surgical specimen to the pathologist for microscopic analysis must provide accurate clinical and surgical information in order to make a definitive diagnosis [4]. The microscopic appearance of a circumscribed but not encapsulated aggregate of mature adipocytes with large clear cytoplasm in the absence of vascularity, atypia or metaplasia is diagnostic of a classical lipoma.

## Conclusion

Buccal soft tissue lipomas are rare tumors. A high index of suspicion is required in making a diagnosis. Surgical excision is the ideal treatment with excellent outcome. The importance of histological diagnosis cannot be overemphasized and the features of lipoma are usually straightforward and classical.

## Consent

Written informed consent was obtained from the patient for publication of this case report and any accompanying images. A copy of the written consent is available for review by the Editor-in-Chief of this journal.

## Competing interests

The authors declare that they have no competing interests.

## Authors' contributions

AAA was the principal surgeon, performed the literature search and prepared the manuscript. TLN assisted in the surgery and postoperative management of the patient. ANM interpreted the slides and reviewed the manuscript. GOE prepared the slides and the photomicrographs of the specimen.
